# Scrotal Size Index: a novel anatomical classification of scrotal morphology in inflatable penile prosthesis surgery

**DOI:** 10.1093/sexmed/qfag067

**Published:** 2026-07-31

**Authors:** Onder Canguven, Ahmed Al Saeedi, Khalid Al Kubaisi, Ahmad Majzoub, Haitham Elbardisi

**Affiliations:** Department of Urology, Hamad Medical Corporation, 3050, Doha, Qatar; Department of Urology, Faculty of Medicine, Istanbul Aydın University, 34295, Istanbul, Turkey; Department of Urology, Hamad Medical Corporation, 3050, Doha, Qatar; Department of Urology, Hamad Medical Corporation, 3050, Doha, Qatar; Department of Urology, Hamad Medical Corporation, 3050, Doha, Qatar; Weill Cornell Medicine Qatar, 24144, Doha, Qatar; Department of Urology, Hamad Medical Corporation, 3050, Doha, Qatar; Weill Cornell Medicine Qatar, 24144, Doha, Qatar

**Keywords:** penile prosthesis complications, prosthetic urology, pump positioning, Scrotal Size Index, scrotal anatomy

## Abstract

**Introduction:**

Pump-related complications remain a significant cause of dissatisfaction and reoperation following inflatable penile prosthesis (IPP) implantation. Despite their clinical importance, scrotal anatomy is currently assessed subjectively, without a standardized method to guide pump placement and surgical decision-making. This study aimed to develop a simple, objective classification system for scrotal morphology and to evaluate its association with intraoperative surgical decisions and pump-related outcomes following IPP surgery.

**Methods:**

A retrospective cohort study was conducted including patients who underwent primary 3-piece IPP implantation via a penoscrotal approach between 2018 and 2024. Dependent scrotal length was measured intraoperatively using a ruler from the penoscrotal junction to the most dependent point of the empty scrotum and recorded for all patients. Based on these measurements, patients were categorized according to the proposed Scrotal Size Index (SSI). Associations between SSI categories, intraoperative surgical variables, and pump-related outcomes were analyzed. Primary outcomes included the relationship between SSI categories and operative variables, including cylinder length, rear tip extender (RTE) utilization, and the need for intraoperative pump- or tubing-related adjustments. Secondary outcomes included pump-related complications within 12 months, including malposition, migration, patient-reported accessibility difficulties, and revision surgery.

**Results:**

A total of 210 patients were included. The mean dependent scrotal length was 7.25 ± 1.56 cm. Distribution analysis demonstrated distinct clustering of measurements (*P* < .001), supporting categorization into 3 anatomical groups: Small (<6 cm), medium (6-9 cm), and large (>9 cm), corresponding to the proposed SSI. The distribution of patients across these categories was 26.2%, 57.1%, and 16.7%, respectively. Significant differences in IPP-related variables were observed among SSI categories. Mean cylinder length increased from 16.2 ± 1.6 cm in the small group to 18.1 ± 1.7 cm in the medium group and 21.9 ± 1.9 cm in the large group (*P* < .001). Mean RTE length similarly increased from 0.9 ± 1.0 cm to 2.1 ± 1.1 cm and 3.0 ± 1.2 cm, respectively (*P* < .001), with corresponding increases in RTE utilization (32.7%, 65.0%, and 82.9%; *P* < .001).

**Conclusion:**

Scrotal morphology appears to be associated with prosthesis-related operative variables during IPP implantation. The proposed SSI offers a simple and reproducible framework for objective anatomical assessment and may assist intraoperative planning. Further prospective studies are required to determine its utility in predicting pump-related outcomes and to establish its clinical validity.

## Introduction

Inflatable penile prosthesis (IPP) implantation remains the definitive treatment for men with erectile dysfunction refractory to medical therapy, with consistently high long-term patient satisfaction rates. Despite these favorable outcomes, device-related complications continue to occur, and reoperation remains an important clinical concern, particularly in the long-term follow-up period.^1^^,^^2^

Among these complications, pump-related issues represent a significant and often underrecognized cause of patient dissatisfaction and surgical revision.^3–7^ The scrotal pump is the functional interface of the device, and its position, accessibility, and stability directly influence ease of use and overall patient experience. Suboptimal pump positioning may result in difficulty with device cycling, discomfort during sexual activity, cosmetic concerns, and, in some cases, the need for reoperation.^3^ Previous studies have demonstrated that technical factors contribute substantially to prosthesis revision, and within this category, pump malposition is a frequent indication for surgical correction.^1^^,^^3^ In particular, postoperative migration of the pump to a high or poorly accessible scrotal position has been identified as a common mechanism leading to functional impairment and patient dissatisfaction.^3^ Optimal pump placement is anatomically dependent. The pump is ideally positioned within the midline scrotal septum in the subdartos space, allowing for stability, concealment, and ease of access.^3^ However, achieving this position is highly influenced by individual patient anatomy. In clinical practice, scrotal morphology varies considerably between patients, affecting available space, depth, and support for the pump.

Despite its clinical importance, scrotal anatomy is currently assessed subjectively, using qualitative descriptors such as “tight,” “adequate,” or “pendulous,” without a standardized or reproducible measurement system. This lack of objective assessment limits surgical planning and may contribute to variability in pump positioning and postoperative outcomes. Furthermore, although modern IPP devices have undergone significant technological refinement, device dimensions-particularly pump size-are relatively consistent across manufacturers, suggesting that anatomical factors may play a more dominant role in determining final pump position than device-specific characteristics.

Given these considerations, there is a clear need for a simple, objective, and clinically applicable method to quantify scrotal anatomy and to better understand its impact on pump positioning and surgical outcomes. Therefore, this study evaluated the role of scrotal morphology in IPP surgery and its association with intraoperative decision-making and early pump-related outcomes in patients undergoing primary implantation. Specifically, can dependent scrotal length be used to develop a simple and reproducible anatomical classification system associated with clinically relevant differences in surgical decision-making and pump-related outcomes following IPP implantation?

## Material and methods

This retrospective observational cohort study was conducted at a tertiary referral center. Institutional approval was obtained from the Medical Research Center (Hamad Medical Corporation; MRC-01-25-1482), and the study was performed in accordance with the Declaration of Helsinki. Given the retrospective design and use of de-identified data, the requirement for informed consent was waived by the Institutional Review Board. Written informed consent for clinical photography and publication of intraoperative images was obtained from all patients prior to surgery.

A total of 210 consecutive adult male patients who underwent primary 3-piece IPP implantation between January 2018 and December 2024 were included. Patients undergoing revision surgery, implantation of malleable or 2-piece prostheses, or those with incomplete intraoperative documentation were excluded.

Scrotal morphology was assessed intraoperatively using a standardized measurement technique. Under sterile conditions and anesthesia, dependent scrotal length was measured using a sterile ruler from the penoscrotal junction to the most dependent point of the empty scrotum. This measurement was routinely documented in operative reports and was used as the primary anatomical variable for analysis. Corporo-pump distance (CPD) is defined as the anatomical distance from the inferior corporotomy (tubing exit point) to the pump tubing entry site within the scrotum, representing the functional relationship between scrotal morphology and tubing configuration. In the present study, CPD was not directly measured intraoperatively; instead, it was retrospectively approximated from operative documentation, incorporating recorded pump position and tubing configuration, and was used as a hypothesis-generating surrogate parameter rather than a formal quantitative variable.

All procedures were performed using a standard penoscrotal approach. Intraoperative variables recorded included prosthesis brand, cylinder length, use and length of rear tip extenders (RTEs), pump pocket creation technique, and final pump position as assessed by the operating surgeon.

Postoperative outcomes were obtained from follow-up records. Pump-related outcomes included malposition, migration, patient-reported accessibility issues, and the need for revision surgery within 12 months.

Data were extracted from electronic medical records using a structured data collection form and anonymized prior to analysis.

Statistical analyses were performed using IBM SPSS Statistics version 26.0 (IBM Corp.). Continuous variables were summarized as mean ± SD, and categorical variables as frequencies and percentages. Given the exploratory nature of the study and the low number of pump-related events, the analysis was primarily descriptive. Group comparisons across SSI categories were performed using chi-square or Fisher’s exact tests for categorical variables and analysis of variance or Kruskal-Wallis tests for continuous variables, as appropriate. Multivariable regression analysis was not performed.

## Results

**Figure 1:**
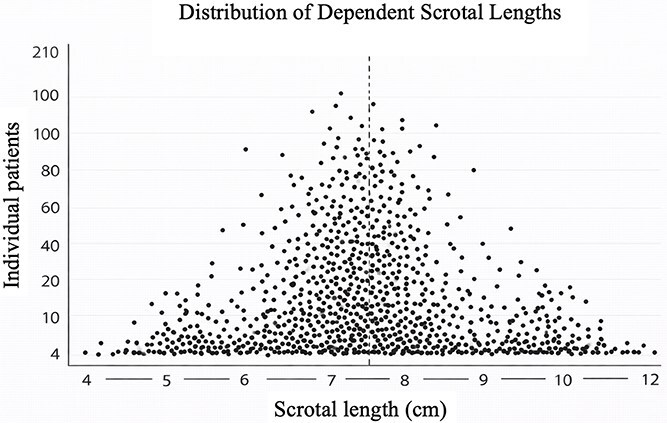
Dot plot showing the distribution of dependent scrotal length across the cohort (*n* = 210), demonstrating substantial anatomical variability and clustering into distinct ranges.

A total of 210 patients undergoing primary IPP implantation via a penoscrotal approach were included in the analysis. Dependent scrotal length was measured intraoperatively in all cases, with a mean value of 7.25 ± 1.56 cm, indicating substantial anatomical variability. Visual inspection of the distribution demonstrated clustering into distinct anatomical ranges (Figure 1).

**Figure 2:**
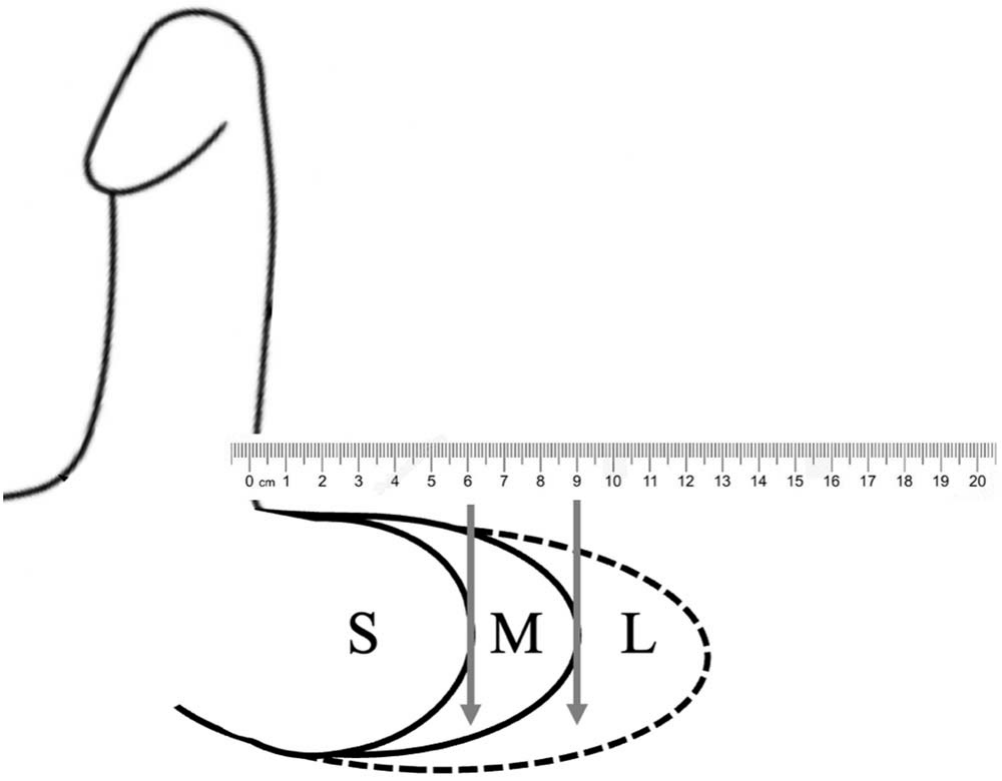
Measurement of dependent scrotal length and Scrotal Size Index (SSI) classification. Dependent scrotal length was measured intraoperatively from the penoscrotal junction to the most dependent point of the empty scrotum using a ruler. Based on this measurement, scrotal morphology was categorized into 3 groups: Small (<6 cm), medium (6-9 cm), and large (>9 cm). The schematic illustrates representative anatomical configurations corresponding to each category.

Based on these findings, scrotal morphology was categorized into 3 clinically relevant groups—small (<6 cm), medium (6-9 cm), and large (>9 cm)—corresponding to the Scrotal Size Index (SSI) (Figure 2). The distribution of SSI categories demonstrated a predominance of medium scrotal morphology (57.1%), followed by small (26.2%) and large (16.7%) groups. Mean scrotal lengths were 5.5 ± 0.4 cm, 7.4 ± 0.8 cm, and 9.8 ± 0.6 cm for the small, medium, and large groups, respectively, demonstrating clear anatomical separation between categories (*P* < .001).

Comparative analysis across SSI categories demonstrated significant differences in IPP-related variables (Table 1). Mean cylinder length increased progressively from 16.2 ± 1.6 cm in the small group to 18.1 ± 1.7 cm in the medium group and 21.9 ± 1.9 cm in the large group (*P* < .001). Similarly, mean RTE length increased from 0.9 ± 1.0 cm to 2.1 ± 1.1 cm and 3.0 ± 1.2 cm, respectively (*P* < .001). The proportion of cases requiring RTE use was significantly higher in larger SSI categories (32.7% vs 65.0% vs 82.9%, *P* < .001).

**Table 1:** Comparison of anatomical and operative variables across Scrotal Size Index (SSI) categories.

Variable	Small (<6 cm) (*n* = 55)	Medium (6-9 cm) (*n* = 120)	Large (>9 cm) (*n* = 35)	*P*-value
Scrotal length (cm)	5.5 ± 0.4	7.4 ± 0.8	9.8 ± 0.6	<.001
Cylinder length (cm)	16.2 ± 1.6	18.1 ± 1.7	21.9 ± 1.9	<.001
RTE length (cm)	0.9 ± 1.0	2.1 ± 1.1	3.0 ± 1.2	<.001
Any RTE use, *n* (%)	18 (32.7%)	78 (65.0%)	29 (82.9%)	<.001
Pump malposition, *n* (%)	1 (1.8%)	1 (0.8%)	1 (2.9%)	—^a^

Continuous variables are presented as mean ± SD and compared using ANOVA or Kruskal-Wallis tests. Categorical variables are presented as percentages and compared using chi-square or Fisher’s exact tests.

^a^Statistical comparison not performed due to low event count.

Abbreviation: ANOVA, analysis of variance; RTE, rear tip extender.

Pump malposition was observed in 3 cases (1.4%) during the 12-month follow-up period. Due to the low number of events, further statistical comparison across SSI categories and multivariable analysis were not performed.

**Figure 3:**
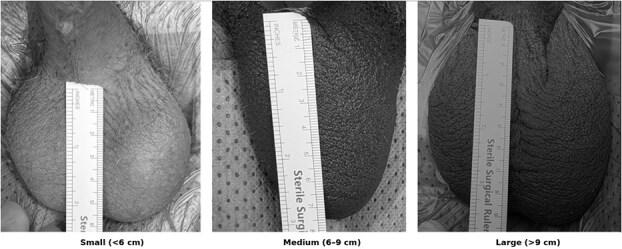
Representative intraoperative photographs demonstrating measurement of dependent scrotal length from the penoscrotal junction to the most dependent point of the scrotum using a sterile ruler. Based on this measurement, scrotal morphology is categorized as small (<6 cm), medium (6-9 cm), and large (>9 cm).

**Figure 4:**
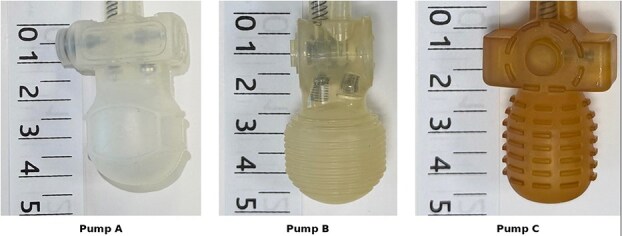
Comparison of pump dimensions across commonly used IPP systems. Representative images of 3 commonly used IPP pumps demonstrate similar overall dimensions, with approximate lengths of 4.5 cm across devices. This highlights that anatomical variability, rather than device size, is the primary determinant of pump positioning and accessibility. IPP, inflatable penile prosthesis.

Representative intraoperative images demonstrating measurement of dependent scrotal length are presented in Figure 3, while representative images of commonly used IPP pumps demonstrate comparable overall dimensions across devices (approximately 4.5 cm), supporting the consistency of device characteristics (Figure 4).

## Discussion

The present study introduces a simple, objective, and clinically applicable method for quantifying scrotal morphology in patients undergoing IPP implantation. By first demonstrating the natural distribution of dependent scrotal length and its clustering into distinct anatomical ranges, this study establishes a data-driven foundation for classification. The proposed SSI, derived from these observations, provides a structured framework that translates anatomical variability into clinically meaningful categories. Importantly, SSI was associated with consistent and significant differences in intraoperative decision-making, including cylinder selection and RTE utilization, supporting its relevance in surgical planning.

Pump-related complications represent a significant and often underrecognized challenge in prosthetic urology. Prior studies have consistently demonstrated that pump malfunction and malposition are major contributors to revision surgery. Cayan et al. reported that among patients undergoing revision procedures, 57% were attributable to pump failure, corresponding to approximately 3% of the total cohort.^1^ Similarly, Mirheydar et al., in a large population-based analysis of 7666 cases, found that mechanical failure accounted for 54% of reoperations, with more than half related to pump malfunction and an overall reoperation rate reaching 15% at 10 years.^8^

Other series have reinforced these findings. Bozkurt et al. reported that approximately 36% of revision surgeries involved pump-related problems, including both failure and erosion.^4^ Chan et al. demonstrated that technical causes accounted for nearly 30% of reoperations, with approximately 40% of these related to pump issues, most commonly postoperative migration.^5^ Importantly, pump malposition has also been associated with chronic postoperative pain that resolved only after repositioning.^5^ Collectively, these data underscore that pump-related complications are not isolated events but represent a fundamental limitation of current IPP surgery.^1^^,^^4^^,^^5^^,^^8^

Despite this clinical relevance, pump positioning remains largely dependent on subjective intraoperative judgment, without a standardized anatomical framework to guide decision-making.^7^^,^^9^ The present study addresses this gap by introducing an objective and reproducible method for classifying scrotal morphology based on dependent scrotal length. Although the present findings suggest that scrotal anatomy may influence pump-related surgical planning, this relationship should be considered hypothesis-generating because pump position was not directly measured. Conceptually, small scrotums may provide less dependent space for pump placement, potentially increasing the likelihood of a higher or less accessible pump position. Conversely, larger scrotums may provide greater dependent depth but could also predispose to greater variability in final pump location, including potential posterior displacement. The intermediate SSI category may represent the most favorable anatomical configuration for pump placement, although this remains to be confirmed in prospective studies. A potential concern when introducing a novel classification system is the selection of cutoff thresholds. In the present study, the categorization of scrotal morphology was both data-driven and clinically grounded. Intraoperative measurements demonstrated clear stratification of scrotal length, with statistically significant separation between groups (*P* < .001) and corresponding differences in prosthesis-related variables, including cylinder length and RTE utilization.

The SSI boundaries (<6 cm, 6-9 cm, and >9 cm) were selected based on the observed distribution of measurements and their apparent intraoperative relevance. Although formal clustering or receiver operating characteristic (ROC)-based methods were not employed, these thresholds provided practical separation of patients into clinically distinct anatomical groups with significantly different operative characteristics. Each SSI category was associated with clinically relevant differences in scrotal morphology and intraoperative decision-making. From a conceptual anatomical perspective, smaller scrotums may provide less dependent space for pump placement, whereas larger scrotums may allow greater dependent depth but could also result in greater variability in final pump location. The intermediate SSI category may represent a favorable anatomical configuration for pump placement (Table 2). These proposed anatomical relationships are hypothesis-generating and require prospective validation using objective measurements of pump position and CPD. From a practical standpoint, these cut-offs align closely with the surgeon’s intraoperative perception of available scrotal space and allow immediate clinical interpretation without the need for complex calculations. While alternative thresholds could be proposed, the present categorization achieves a balance between anatomical discrimination and clinical usability and provides a reproducible foundation for standardizing scrotal assessment in prosthetic surgery. Future prospective studies may further refine the optimal SSI cutoff values and assess their predictive performance. In this respect, SSI may serve a role analogous to other clinically adopted anatomical indices, such as body mass index, by providing a standardized and reproducible framework for patient stratification.

**Table 2:** Conceptual schematic illustrating the proposed anatomical relationships between Scrotal Size Index (SSI), corporo-pump distance (CPD), and potential pump positioning.

SSI parameter	Category	Anatomical meaning	Conceptual pump position	Conceptual CPD	Operative comments
**Supine scrotal drop length**	Small (<6 cm)	High, tight scrotum with minimal dependent drop	Mostly anterior/high	<3 cm	Prevents high-riding pump; limited pocket space
	Medium (6-9 cm)	Average scrotal depth; balanced geometry	Dependent/midline	3-6 cm	Ideal anatomy for pump seating and tubing routing
	Large (>9 cm)	Deep, pendulous scrotum	Posterior descent likely	>6 cm	Risk of posterior migration; ensure adequate tubing slack

CPD ranges and pump-position descriptors presented in this table are conceptual and are based on anatomical reasoning and surgical experience. They were not directly measured in the present study and should not be interpreted as validated quantitative thresholds.

Corporo-pump distance is proposed as a hypothesis-generating anatomical concept that may help describe the relationship between scrotal morphology, tubing configuration, and final pump location. Because CPD was not directly measured and was retrospectively inferred from operative documentation, it should be interpreted as a conceptual model rather than a validated anatomical parameter. Future prospective studies incorporating direct intraoperative measurement of CPD will be necessary to determine its reproducibility, anatomical validity, and potential clinical relevance.^7^^,^^9^

Importantly, our findings also suggest that device-related factors play a limited role in pump positioning variability. Comparative evaluation of commonly used IPP pumps demonstrated similar physical dimensions across manufacturers, supporting the concept that anatomical variation, rather than device design, is the dominant determinant of pump positioning outcomes.^3^^,^^6^^,^^9^

From a surgical perspective, these findings reinforce established principles of optimal pump placement. The pump should ideally be positioned within the midline subdartos space, ensuring accessibility while avoiding excessively superficial placement, high positioning near the penile shaft, or low displacement toward the perineum.^7^^,^^9^^,^^10^ Inadequate positioning may result in cosmetic dissatisfaction, discomfort, erosion, or functional impairment.^3^^,^^5^ Proper tubing management and fixation are also critical to prevent migration and ensure long-term stability.^3^^,^^7^

Patient-reported outcomes further emphasize the importance of pump-related factors. Habous et al. demonstrated that even among patients with intermediate satisfaction, approximately 30% reported pump-related issues, highlighting that pump function and accessibility are key determinants of overall satisfaction and should not be underestimated.^2^

This study has several limitations. The retrospective design introduces inherent limitations, including potential selection bias and reliance on recorded measurements. CPD was not directly measured and was retrospectively approximated from operative documentation; therefore, it was used as a hypothesis-generating conceptual framework rather than a validated anatomical parameter. Furthermore, the SSI classification was derived post hoc based on observed clustering of scrotal measurements and their apparent surgical relevance. Although SSI demonstrated associations with intraoperative decision-making variables, the low number of pump-related events, with only 3 cases of malposition observed, precluded formal assessment of its predictive performance and does not allow definitive validation of the classification system. Consequently, SSI should be considered a hypothesis-generating anatomical framework that requires prospective external validation in larger cohorts with sufficient clinical outcome events.

Follow-up was limited to early postoperative outcomes, and longer-term studies are needed to determine whether SSI predicts clinically meaningful endpoints such as pump accessibility, patient satisfaction, pump malposition, and revision surgery. Additionally, all procedures were performed using a penoscrotal approach, which provides procedural consistency but may limit generalizability to other surgical techniques. Finally, the low event rate prevented robust multivariable analyses. Therefore, we cannot exclude the possibility that factors such as age, body mass index, diabetes mellitus, prior pelvic surgery, or IPP characteristics may have contributed to the observed associations. Future prospective studies with larger cohorts and sufficient clinical outcome events should evaluate whether SSI remains independently associated with pump-related outcomes after adjustment for these potential confounders.

Despite these limitations, this study introduces the first objective and clinically applicable framework for quantifying scrotal morphology in IPP surgery. SSI offers a simple, reproducible, and intuitive method for anatomical assessment that may facilitate surgical planning and provide a standardized foundation for future investigations evaluating the relationship between scrotal anatomy, pump positioning, and patient outcomes.

## Conclusion

The SSI provides a simple, objective, and clinically applicable framework for quantifying scrotal morphology in IPP surgery. By translating anatomical variability into standardized categories, SSI may facilitate intraoperative decision-making and provide a foundation for future studies evaluating the relationship between scrotal anatomy and pump-related outcomes.
